# Study on thermal insulation and seismic effect for fault-crossing tunnel in high geothermal area

**DOI:** 10.1038/s41598-022-15626-4

**Published:** 2022-07-05

**Authors:** Guangyao Cui, Wenhao Shi

**Affiliations:** School of Civil Engineering, North China University of Technology, No.5 Jinyuanzhuang Road, Beijing, 100144 China

**Keywords:** Civil engineering, Natural hazards

## Abstract

The fault-crossing tunnel in high geothermal areas can be severely damaged under strong earthquake. In this paper, numerical models were established in Flac^3d^ to investigate the thermal insulation and seismic effects under the strong earthquake when using highly damped composite structure. The results showed that the thermal insulation effect of the PC, the PC highly damped composite structure and the SFRC highly damped composite structure were almost the same, which the PC highly damped composite structure was slightly better. After applying the highly damped composite structure, the maximum and minimum principal stresses on the lining decreased, it showed that highly damped composite structure could enhance the seismic effect of tunnel. The safety factor of SFRC highly damped composite structure was 2.420, which was a 28.14% increase compared to PC. Based on numerical simulations, the thermal insulation and seismic effect of SFRC highly damped composite structure was better than other seismic measures. The research can provide a reference for the design of thermal insulation and anti-earthquake for fault-crossing tunnel under strong earthquake in high geothermal area.

## Introduction

Seismic activity is frequent in the western area of China, high intensity earthquake will lead to serious geological hazards and damage the stability of buildings and transportation network^1^. Tunnels occupy an extremely important place in ensuring normal and convenient traffic in mountainous areas of China, damage to them caused by earthquakes and faults can seriously affect the transportation network, so it is essential to ensure their safety and durability^2^. Although underground buildings typically suffer a lower rate of damage than buildings on the ground in a natural disaster, tunnels will also take significant damage under strong earthquake. For tunnels located in complex geological environments, their design of safety must be considered carefully.

High intensity earthquake can cause fault misalignment, although the duration of it is relatively short during the earthquake, the generated repeated shear and compression of the surrounding rock fragmentation and misalignment will produce irreversible damage to the main and portal structure of tunnel^3,4^. The fault movement might be divided into fault movement and seismic motion under the strong earthquake action, and both of them could make a huge impact on the stability of tunnel structure^5^. According to the investigation of historical strong earthquake events, the fault of the tunnel is the most vulnerable part of the tunnel during strong earthquake, including the 1999 Chi-Chi, the 2004 Mid-Niigata, the 2008 Wenchuan and the 2016 Kumamoto earthquake^6–8^. As the most common adverse geological phenomenon in tunnel construction, the area where the fault exists is one of the most unstable areas of the surrounding rock, it may lead to tunnel collapse, large deformation and other construction geological hazards. The impact of faults on tunnel construction is mainly manifested in reducing the overall strength of the surrounding rock and changing the physical and mechanical properties of the rock mass. The presence of faults significantly reduces the strength of the tunnel structure, and the damage instability of the tunnel structure is mainly caused by the shear strength of the weak structural surfaces formed by the fault zone. The strength of these structural surfaces is generally very low, and the deformation resistance and deformation modulus are also lower than those of the rock material, this leads to low strength of the surrounding rock in the fault area^9^. The section of the fault zone is the key paragraph for the seismic design of the tunnel.

The researches about tunnel engineering are usually carried out with the help of numerical simulation. In current numerical study, continuum and dis-continuum based numerical simulations are two major numerical techniques in tunnel engineering^10^, which continuum modeling is more commonly used. The continuum-based methods contain finite element method (FEM)^11^, finite difference method (FDM)^12,13^, extended finite element method (XFEM)^14,15^ and meshless method (MM)^16–18^, etc. The thermal analysis is necessary for high geothermal tunnel, since the high ground temperature would cause damage to workers’ health, reduction in the efficiency of site operations, destruction of tunnel structures, failures of mechanical equipment, and increases in accident rates^19–23^. The temperature stress on the lining structure would reduce the safety of initial support and second lining^24–26^. Thus, the previous studies are concentrated on the analysis of lining temperature and its mechanical property: Lin et al.^27^ studied the temperature field and the temperature variation of lining structure under different cooling measures by numerical simulation. Liu et al.^28^ presented the mechanical behavior of high geothermal tunnel lining. Wang et al.^29,30^ studied the mechanical properties of cement-based grouting materials used in high geothermal area tunnels and provided recommendations for their compressive constitutive design. Cui et al.^31^ investigated the effect of hot-humid and hot-dry environments on the deformation of shotcrete in the context of high geothermal tunnels. Liu et al.^32^ set several tests to analyze the mechanical properties and pore structure characteristics of shotcrete under hot-dry environments at different temperature.

However, with the development of tunnel construction in the mountainous areas of western China, there have been cases where the tunnels are located in the fault zone and the high geothermal area at the same time. Previous studies have focused on one of the fault-crossing tunnels and high geothermal tunnels, while less research about the combination of the above two. Flac^3d^ software is a finite difference software, it is used dramatically in the study of dynamic response and thermal analysis of tunnel. In this paper, taking the Sangzhuling tunnel section of the Sichuan-Tibet railway line as background, a numerical model was established in Flac^3d^ to study the difference of thermal insulation and seismic effect when taking PC, PC highly damped composite structure, SFRC and SFRC highly damped composite structure as seismic measures. The results can provide a reference for the design of tunnels crossing faults under strong earthquake in high geothermal area.

## Materials and methods

### Simulation method

Using Flac^3d^ to the establish and analyze the model in the study, Flac^3d^ (Fast Lagrangian Analysis of Continua in 3Dimensions) is an extend program of Flac^2d^^33^. It is a three-dimensional and explicit finite difference program, and it uses the mixed discretization procedure to study the mechanical behavior of three-dimensional structures built of soil, rock or metal materials that may occur plastic flow when their yield limits are reached^34^. The basic principle of the mixed discretization procedure is similar to the discrete element method, but it is more applicable to solve continuous problems in irregular regions with various material modes and boundary conditions^35^. Flac^3d^ uses an explicit time-marching solution where each polyhedral element of the model responds according to a prescribed linear and/or nonlinear constitutive law in response to applied forces or kinematic boundary conditions^36^.

In Flac^3d^, the explicit finite difference method is used in the dynamic analysis process, its unique dynamic-multi-stepping method can effectively reduce the required time of solving^37^, and keep numerical scheme staying stable even when the physical system may be unstable. It contains six forms of unit structure in Flac^3d^: beam, cable and pile are three kinds of linear structure; shell, geogrid and liner are three kinds of shell structure. Each unit consists of nodes and structural elements. By linking nodes with zone or other nodes, it can simulate the simulate of soil or structure with structural elements. By defining the material parameters, earthquake motion, boundary conditions in the software, the seismic response of tunnel structures under earthquake can be accurately simulated.

### Numerical model

**Figure 1:**
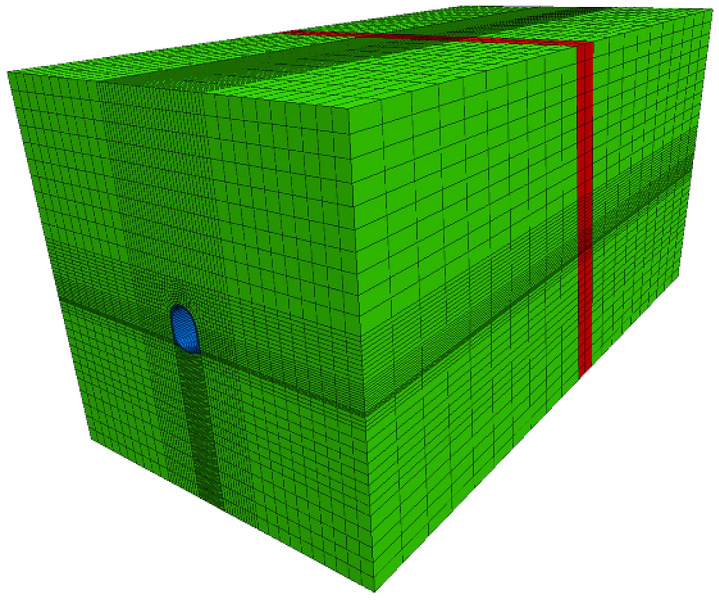
Numerical model of Sangzhuling Tunnel.

The elastic–plastic model was established to simulate the thermal insulation and seismic effect of Sangzhuling fault-crossing tunnel under strong earthquake in high geothermal area. The height of model was 105 m and the longitudinal length was 150 m, the height, width, and burial depth of tunnel was 15, 10 and 45 m respectively. In order to eliminate the influence of boundary effects, the left and right width of the model was taken as 5 times the width of the tunnel, which was 50 m. The thickness of the bedrock at the bottom was 20 m. The dip angle of the fault zone was about 80°, and the width was 10 m. The model was meshed to divide the model into a finite number of elements which were interrelated and mutually binding and to perform numerical simulations in Flac^3d^. The width and height of the surrounding rock mesh size were both 4 m, and those of the rock mesh size near the tunnel were both 1 m. The hanging wall and footwall was longitudinal divided into 10 equal parts, and the fault zone was divided into 2 parts. The overall model is shown in Fig. 1.

**Figure 2:**
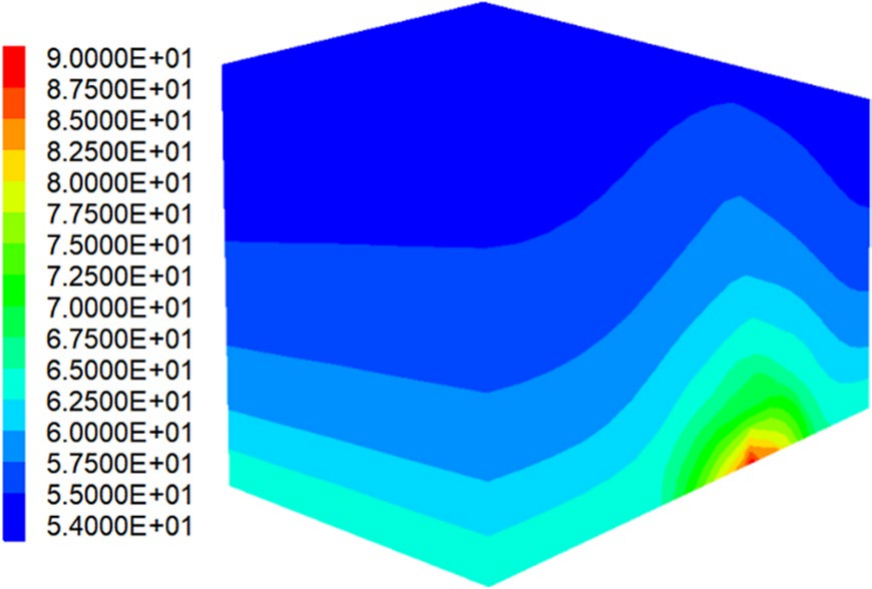
Cloud map of temperature distribution.

To study the thermal insulation effect of tunnel, it needs to set the temperature field in the program. According to the geological exploration report, the temperature of the fault zone about 100 m below the tunnel is close to 90 ℃, we set it as the temperature of the fault zone at the bottom of the model in this study. After transmitting upwards from the bottom of the fault, the temperature at the top of the model were 54 ℃. The stable temperature distribution is shown in Fig. 2.

### Test cases

**Figure 3:**
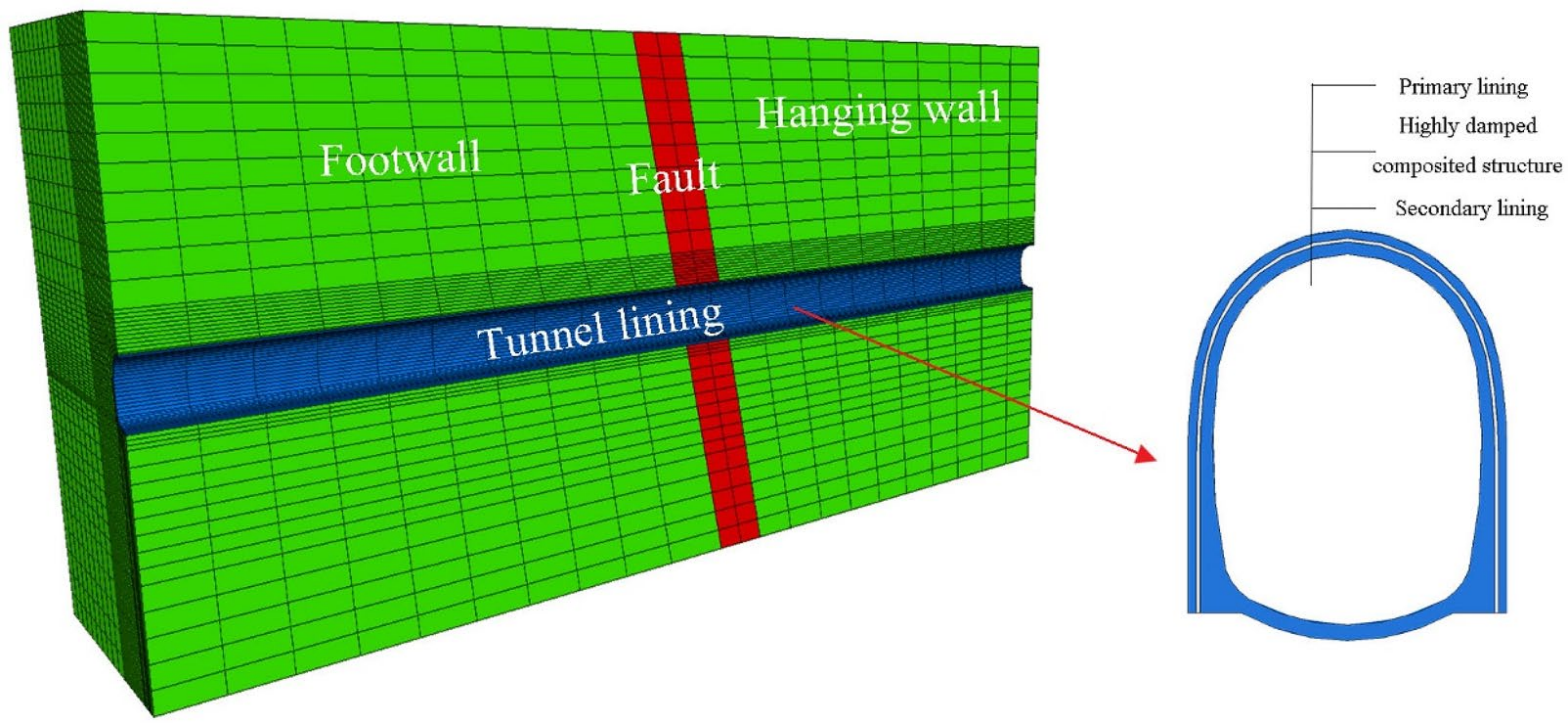
Application method of highly damped composite structure.

A total for four test cases were taken to analyze the difference of thermal insulation and seismic effect under different seismic measures. Plain concrete and steel fiber concrete were used as different lining materials for comparative analysis. The highly damped composite structure was applied between the primary lining and the second lining, as shown in Fig. 3. The test cases of this paper are listed in Table 1.

**Table 1:** Test cases.

Test cases	Seismic measure	Thickness of highly damped composite structure
1	Plain concrete (PC)	–
2	PC highly damped composite structure	9 mm
3	Steel fiber reinforced concrete (SFRC)	–
4	SFRC highly damped composite structure	9 mm

### Geological condition

Sangzhuling tunnel of Sichuan-Tibet railway is 16449 m long, which is one of the important railroad projects in the west of China. The tunnel is designed and constructed using the New Austrian method, with curved wall lining and composite lining structure. The primary lining is C20 shotcrete with a thickness of 0.25 m, and the second lining is C25 modeled concrete with a thickness of 0.45 m. The tunnel is located in the Yarlung Tsangpo River suture zone, where the Indian plate collides with the Eurasian plate, the environment around the tunnel exists strong and frequent geothermal activity. As adverse geology such as high soil temperature and hot water are especially common near the tunnel, the maximum surrounding rock temperature of the tunnel can reach 90 °C and the ambient temperature inside the tunnel can reach up to 56 °C. The tunnel also crosses the Waka graben east fault zone, due to the loose and fragmented surrounding rock of the fault, its high thermal conductivity leads to the heat generated by geothermal activity being easily transferred to the tunnel via the fault. According to the geological survey and current code for design of road tunnel, the surrounding rock of the tunnel are Grade IV rocks, the fault zone surrounding rock are Grade V fragile rocks, and the bedrock are Grade II hard rocks.

### Highly damped composite structure

In tunnel engineering, highly damped materials are usually used in seismic measures for lining reinforcement to decrease the tunnel vibrations caused by earthquakes. The greater the damping effect, the more energy can be dissipated in a shorter period of time. Such as applying a soft layer to cover the tunnel lining to mitigate damage during an earthquake^38^, injecting a kind of soft material into the interspace between the tunnel lining and the surrounding rock to reduce shear forces on the soil-tunnel interface^39^ and employing constrained layer damping treatments in the design of earthquake-resistant structure to absorb seismic energy. In contrast to the above articles, in this paper we studied the difference of thermal insulation and seismic effect when using highly damped composite structure for the two materials lining.

### Material parameters

The parameters of highly damped composite structure used in the study were based on Qingdao Shamu damping Qtech-506, which is a kind of damping coating for the concrete surface of the tunnel. It is a kind of critical material between solid and high viscosity liquid. Under the condition of room temperature (25 ℃), the viscosity is about 6 million centipoises, the damping factor is about 0.51. Since it is a kind of damping material, its hardness is measured by Shore A hardness and the value is 20. The Poisson’s ratio is 0.5, and the elongation at break is greater than 1000%. The material adhesion is 1.65 MPa. The thermal conductivity of damping layer is 0.144 W/m·℃. The parameters of the surrounding rock were obtained from local geological exploration data. The necessary parameters used for the study are listed in Table 2.

**Table 2:** Parameters of the materials.

Name	Density/(kN/m^3^)	Modulus of elasticity/GPa	Poisson’s ratio	Friction angle/°	Cohesion/MPa	Thermal conductivity/(W/m·℃)
Surrounding rock (Class IV)	2200.0	5.0	0.3	35.0	0.5	8.0
Bedrock (Class II)	2500.0	20.0	0.2	50.0	1.5	2.2
Fractured zone (Class II)	2000.0	2.0	0.4	25.0	0.2	10.0
C20 shotcrete	2200.0	23.0	0.2	–	–	2.0
C25 modeled concrete (PC)	2500.0	28.0	0.2	–	–	2.0
C25 modeled concrete (SFRC)	2500.0	29.5	0.2	–	–	2.6
Highly damped composite structure	1100.0	0.3	0.5	5.0	5	0.1

### Dynamic calculation

**Figure 4:**
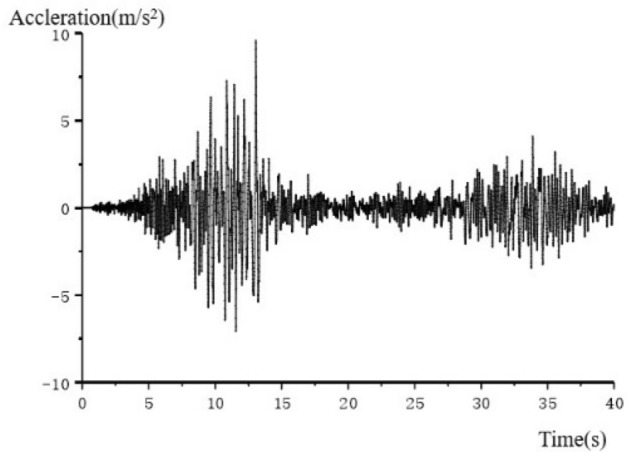
Time history curves of acceleration (x direction).

By means of writing fish language in Flac^3d^, the software can simulate the effects of earthquake on tunnels, and the dynamic loads can be applied directly to the external boundaries of the model. Using the actual seismic wave measured by the Wolong station in 2008 Wenchuan earthquake to simulate the situation that tunnel affected by earthquake. The original seismic waves were filtered and baseline corrected by professional seismic wave processing software, which can reduce the effect of grid size on seismic waves^40^. Seismic waves were transmitted from the bedrock at the bottom of the model to above, and their horizontal, vertical and longitudinal seismic waves correspond to the x, y and z directions of the model respectively, with a duration of 15 s and an acquisition interval of 0.01 s. The local damping factor was 0.1571. Figure 4 shows the time history curves of acceleration of x direction.

### Monitoring system

**Figure 5:**
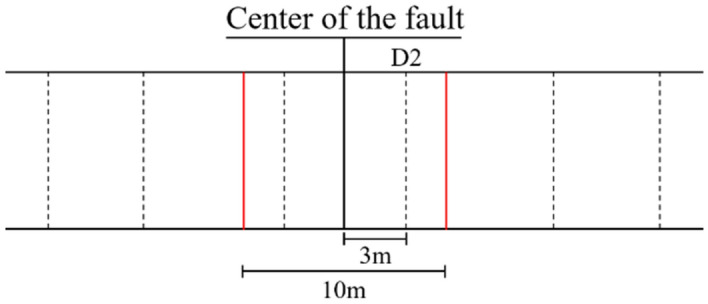
Monitoring section of the tunnel.

**Figure 6:**
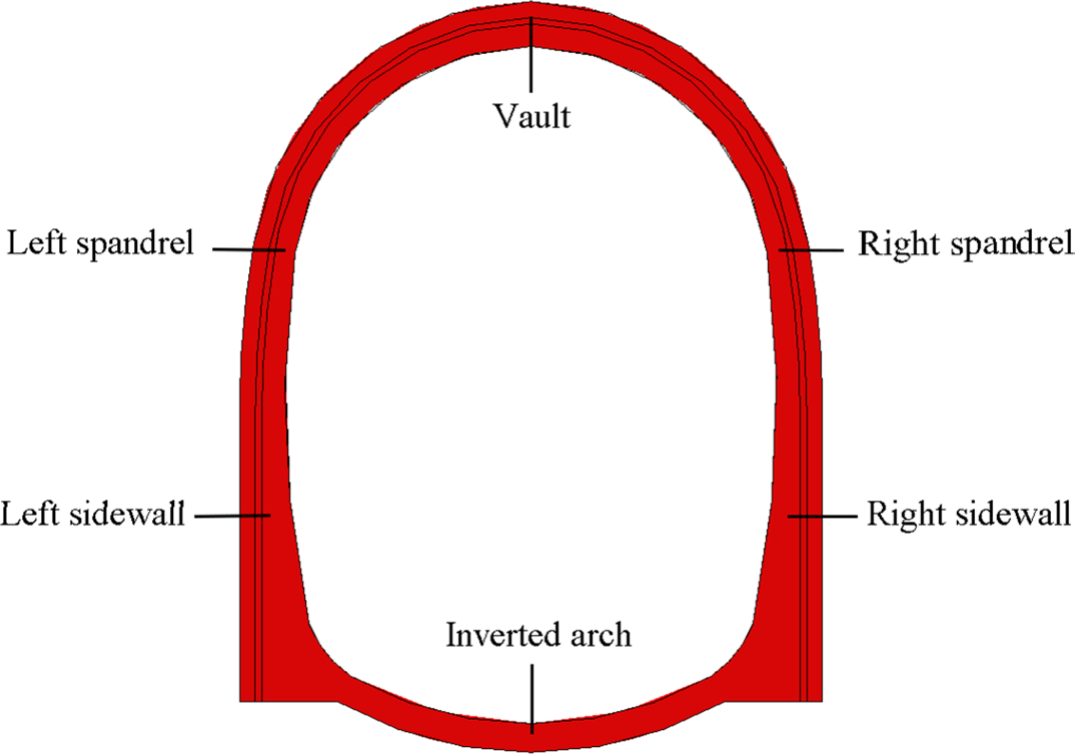
Layout of measuring points.

Flac^3d^ can dynamically monitor data such as the displacement, the stress and strain of each unit and node during the dynamic analysis and export their cloud maps at the end of the analysis. In the study, D2 monitoring section was placed along the vertical direction of the tunnel, so as to collect the stress, temperature, displacement and other data of the tunnel structure in the earthquake. Five measuring points (i.e., vault, left spandrel, right spandrel, left sidewall, right sidewall) were set to collect the internal force data. Figure 5 shows the monitoring section of the tunnel. The layout of measuring points is shown in Fig. 6.

### Consent for publication

If this article is accepted, we agree to transfer the copyright to this journal.

## Results of calculation

As the surrounding rock in the fault zone is loose and fragile and has a high thermal conductivity, the tunnel structure in the fault zone is less safe and most severely affected by high ground temperature, the requirements for thermal insulation and seismic effect of the tunnel structure near the fault is the highest among the whole tunnel. The D2 section was chosen for analysis, which was a cross-fault monitoring section in the footwall.

### Thermal insulation

**Figure 7:**
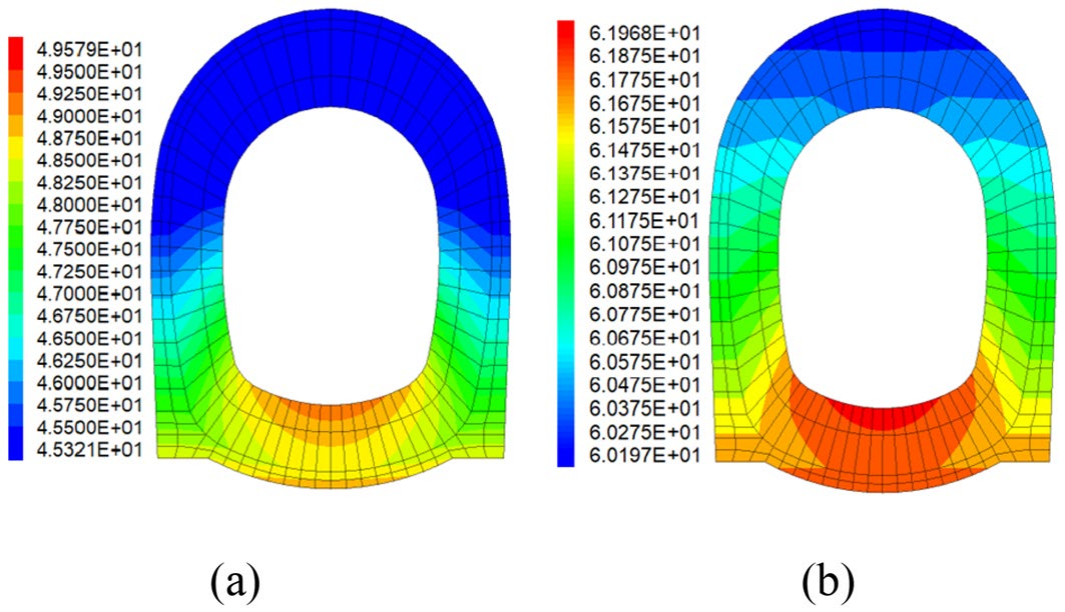
Cloud maps of temperature: (**a**) PC; (**b**) SFRC.

After the numerical simulations, the cloud maps and data of temperature could be output from the Flac^3d^. Figure 7 shows the cloud maps of temperature for PC and SFRC. The maximum temperature values of the second lining under each case are shown in Table 3, and the results of the comparison between each seismic measure are shown in Table 4.

**Table 3:** Maximum temperature value on the right side of D2 section.

Monitoring point	PC/(℃)	PC highly damped composite structure/(℃)	SFRC/(℃)	SFRC highly damped composite structure/(℃)
Vault	45.32	44.78	60.23	45.83
Right spandrel	45.49	44.87	60.27	45.87
Right sidewall	46.89	46.75	60.75	47.76
Inverted arch	49.58	48.82	61.97	49.78

**Table 4:** Comparison of thermal insulation effect on the right side of D2 section.

Monitoring point	Compared with PC			Compared with PC highly damped composite structure	Compared with SFRC
	PC highly damped composite structure	SFRC	SFRC highly damped composite structure	SFRC highly damped composite structure	SFRC highly damped composite structure
Vault	1.19%	− 32.90%	− 1.13%	− 2.34%	23.91%
Right spandrel	1.36%	− 32.49%	− 0.84%	− 2.23%	23.89%
Right sidewall	0.30%	− 29.56%	− 1.86%	− 2.16%	21.38%
Inverted arch	1.53%	− 24.99%	− 0.40%	− 1.97%	19.67%

Since the results of the left and right side of the tunnel was basically the same, the right half of the tunnel was taken as an example for analysis. The data in Table.3 shows that the temperature of the second lining of the tunnel is the highest when using steel fiber concrete, which are around 60 ℃. The thermal insulation effect of the remaining three seismic measures varies, while plain concrete highly damped composite structure is slightly better. It can be seen that the effect of steel fiber concrete is the worst in reducing the temperature of the second lining, and the that of plain concrete highly damped composite structure is the best in the four seismic measures.

The data in Table 4 shows that, when using different lining materials, the thermal insulation effect of SFRC compared to PC is reduced by 24.99–32.90%, and that of SFRC highly damped composite structure compared to PC highly damped composite structure is reduced by 1.97–2.34%, which shows that the thermal insulation effect of PC lining is better than that of SFRC lining.

When using highly damped composite structure, the thermal insulation effect of PC highly damped composite structure is improved by 0.30–1.53% compared to PC, and that of SFRC highly damped composite structure is improved by 19.67–23.91% compared to SFRC. The applying of highly damped composite structure could reduce the temperatures lining markedly, especially for SFRC lining.

After incorporating steel fibers to the concrete, the temperature of SFRC lining is higher than PC lining. Its incorporating leads to higher thermal conductivity of concrete and causes higher temperature of lining. The applying of highly damped composite structure is capable of counteracting the effect of fiber incorporation on thermal conductivity, it reduces the temperature transfer to the free face of tunnel.

### Maximum principal stress

**Figure 8:**
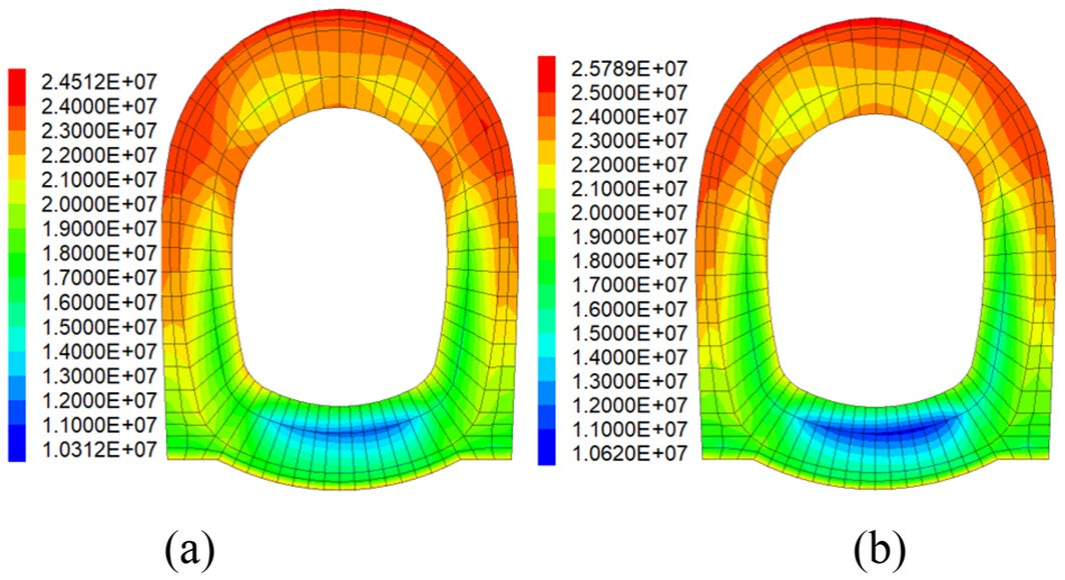
Cloud maps of maximum principal stress: (**a**) PC; (**b**) SFRC.

The maximum principal stresses of each test case were calculated separately. Figure 8 displays the cloud maps of maximum principal stress for PC and SFRC. The calculation results are shown in Table 5.

**Table 5:** Maximum principal stress and seismic effect of section D2.

Monitoring point	PC group			SFRC group		
	PC/(MPa)	PC highly damped composite structure/(MPa)	Seismic effect/(%)	SFRC/(MPa)	SFRC highly damped composite structure/(MPa)	Seismic effect/(%)
Vault	24.51	22.34	8.85	25.78	24.31	5.71
Right spandrel	21.22	15.68	26.02	21.36	20.47	4.30
Left spandrel	23.62	14.63	38.09	23.62	23.23	1.62
Right sidewall	13.72	9.65	29.64	13.53	12.98	4.26
Left sidewall	10.75	9.26	13.81	11.72	10.85	7.31
Invert arch	10.31	8.94	13.29	10.62	9.48	10.73

In PC group, the maximum principal stresses for PC ranges from 10.31 to 24.51 MPa, while those of PC highly damped composite structure ranges from 8.94 to 22.34 MPa. The applying of highly damped composite structure reduced the maximum principal stresses of PC lining by 8.8–38.09%. In FRC group, the maximum principal stresses for SFRC are between 10.62 and 25.78 MPa, and the stresses of SFRC highly damped composite structure are between 9.48 and 24.31 MPa. The applying of highly damped composite structure increases the seismic effect of maximum principal stress for SFRC lining by 1.62–10.73%.

It shows that the highly damped composite structure can absorb earthquake energy, it enhances the seismic effect of maximum principal stress for both PC lining and SFRC lining at all monitoring points. Besides, the maximum principal stresses of SFRC lining are larger than those of PC lining, because the steel fibers incorporated in the concrete increases the stiffness of the lining. Although the maximum principal stresses on the SFRC lining are increased, further calculations are needed to determine their safety.

After applying highly damped composite structure, its capacity of viscous seismic energy dissipation could dramatically reduce the maximum principal stress of lining. The incorporation of steel fibers increases the strength and stiffness of lining. It also enhances the inhibitory effect on the deformation of the surrounding rock, resulting in an increase in the surrounding rock pressure on the lining.

### Minimum principal stress

**Figure 9:**
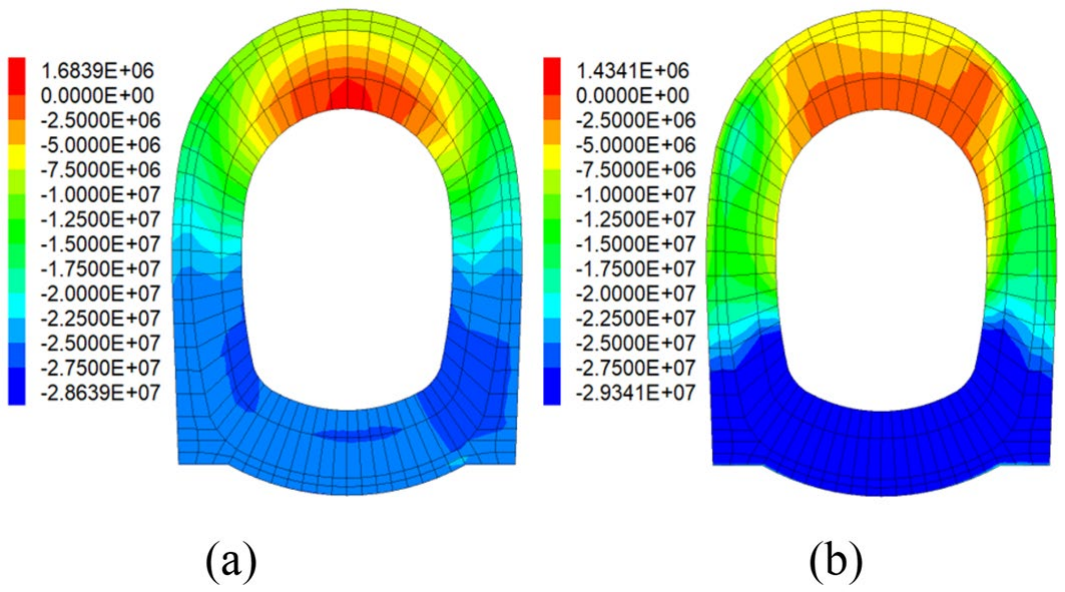
Cloud maps of minimum principal stress: (**a**) PC; (**b**) SFRC.

Similar to the analysis of maximum principal stress, Fig. 9 displays the cloud maps of minimum principal stress for PC and SFRC. The calculation results are shown in Table 6.

**Table 6:** Minimum principal stress and seismic effect of section D2.

Monitoring point	PC group			SFRC group		
	PC/(MPa)	PC highly damped composite structure/(MPa)	Seismic effect/(%)	SFRC/(MPa)	SFRC highly damped composite structure/(MPa)	Seismic effect/(%)
Vault	1.68	1.49	11.31	1.43	1.40	0.90
Right spandrel	− 16.93	− 5.62	66.96	− 8.03	− 7.85	2.23
Left spandrel	− 17.03	− 6.24	63.51	− 8.07	− 7.82	3.22
Right sidewall	− 20.96	− 19.13	8.75	− 26.11	− 19.97	23.63
Left sidewall	− 26.35	− 19.72	25.12	− 27.49	− 22.98	16.60
Invert arch	− 28.64	− 20.01	30.13	− 29.34	− 23.67	19.33

For PC lining, the minimum principal stresses for PC ranges from − 28.64 to 1.68 MPa, and the stresses of the PC highly damped composite structure ranges from − 20.01 to 1.49 MPa, which improved the seismic effect of minimum principal stress by 8.75–66.96%. And for SFRC lining, the minimum principal stresses for SFRC varies between − 29.34 and 1.43 MPa, the minimum principal stresses of SFRC highly damped composite structure varies between − 23.67 and 1.40 MPa, its seismic effects of minimum principal stress are increased by 0.90–23.63% compared to the former.

Both the maximum principal stresses and minimum principal stresses are decreased after using highly damped composite structure in RC and SFRC lining, which shows its stable ability of decreasing the lining stress. The physical properties of damped structure enable it to share the stress from surrounding rock with the lining structure.

The characteristic of viscous seismic energy dissipation of highly damped composite structure is also helpful for reducing the minimum principal stress on lining. After enhancing the strength and stiffness of lining, the maximum and minimum principal stress are both decreased by applying highly damped composite structure.

### Safety factor

Safety factor is used for the design of tunneling structures to reflect the structural safety under seismic measures. Larger safety factor means better safety of the structure. However, related data such as the axial force and the bending moment cannot directly obtained in Flac^3d^. Thence the axial force and the bending moment of lining is calculated according to the Eqs. (1) and (2).

1 $$ \begin{array}{*{20}c} {N = \frac{{E\left( {\sigma_{1} + \sigma_{2} } \right)bh}}{2}} \\ \end{array} $$

2 $$ \begin{array}{*{20}c} {M = \frac{{E\left( {\sigma_{1} - \sigma_{2} } \right)bh^{2} }}{12}} \\ \end{array} $$

where *N* is the axis force, *M* is the bending moment, *E* is the elastic modulus, *σ*_1_ is internal stress of lining, which is calculated by Eq. (3). *σ*_2_ is the external stress of lining, which is calculated by Eq. (4), *b* is the width of the tunnel section, *h* is the thickness of the tunnel section.

3 $$ \begin{array}{*{20}c} {\sigma_{1} = \varsigma \left( {\sigma_{n1} + \sigma_{n2} } \right) + \frac{{\varsigma \left( {\sigma_{n1} + \sigma_{n2} } \right)}}{\xi }} \\ \end{array} $$

4 $$ \begin{array}{*{20}c} {\sigma_{2} = \varsigma \left( {\sigma_{n1} + \sigma_{n2} } \right) - \frac{{\varsigma \left( {\sigma_{n1} + \sigma_{n2} } \right)}}{\xi }} \\ \end{array} $$

where *ς* is the reciprocal of lining zone layers, *σ*_*n*1_ is the normal stress of the internal grid, *σ*_*n*2_ is the normal stress of the external zone, *σ*_*n*1_ and *σ*_*n*2_ are calculated in Eq. (5)

5 $$ \begin{array}{*{20}c} {\sigma_{n} = \sigma_{x} \cos^{2} \left( { - \alpha } \right) + \sigma_{y} \sin^{2} \left( { - \alpha } \right) + \sigma_{xy} \sin \left( { - 2\alpha } \right)} \\ \end{array} $$

where *α* is the angle between the line of centroid of inner and outer elements and vertical direction, which is calculated by Eq. (6); (*x*_1_, *y*_1_) and (*x*_2_, *y*_2_) are the centroid coordinates of the inner and outer zones, respectively.

6 $$ \begin{array}{*{20}c} {\alpha = \arctan \left( {\frac{{x_{2} - x_{1} }}{{y_{2} - y_{1} }}} \right)} \\ \end{array} $$

According to the Code for Design of Railway Tunnel^41^, the safety factors of four seismic measures are calculated by Eqs. (7) and (8). The results are shown in Table 7.

7 $$ \begin{array}{*{20}c} {KN \le \varphi \delta R_{a} bh} \\ \end{array} $$

8 $$ \begin{array}{*{20}c} {KN \le \varphi \frac{{1.75R_{l} bh}}{{\frac{{6e_{0} }}{h} - 1}}} \\ \end{array} $$

where *K* is the safety factor, *φ* is the longitudinal bending coefficient, which takes 1.0 in the study, *δ* is the influence coefficient of axial force eccentricity, *R*_*ɑ*_ is the ultimate compressive strength of concrete, *R*_*l*_ is the ultimate tensile strength of concrete, *e*_0_ is the eccentricity of section.

**Table 7:** Safety factor and seismic effect of section D2.

Seismic measures	Minimum safety factor	Seismic effect/(%)
PC	1.739	–
PC highly damped composite structure	1.820	4.45
SFRC	2.065	15.79
SFRC highly damped composite structure	2.420	28.14

Taking the minimum safety factors of section D2 for analysis, and compared their seismic effects. From Table 7, it shows that the minimum safety factors from PC to SFRC highly damped composite structure are raised gradually, which are 1.739, 1.820, 2.065 and 2.42. Compared to PC, the seismic effects of the other three seismic measures are improved by 4.45, 15.79 and 28.14% respectively. The seismic effect of SFRC highly damped composite structure in safety factor is the best in all seismic measures, which have an increase of 28.14%.

The steel fibers incorporated in the concrete increases the stiffness of the lining, which improves the ability of limiting the deformation of surrounding rock. The steel fibers incorporated in the concrete increases the maximum and minimum principal stress on the lining, but also improves the upper limit of the stress that the lining can withstand. The results indicate that the SFRC lining has better performance in the safety of tunnel lining, even though larger maximum and minimum principal stresses on the lining structure.

In summary, the performance of viscous seismic energy dissipation is exerted after using highly damped composite structure, it reduces the seismic force on the lining and the pressure transmitted from the surrounding rock to the lining, then enlarging the safety factors. However, the increase in safety factors is not obvious when only applying the highly damped composite structure. After using SFRC lining, the strength, stiffness and toughness of lining are increased, it also improved the ability of resisting earthquake forces and the safety of lining. Considering the thermal insulation effect of highly damped composite structure is great while its seismic effect is general, and the SFRC lining has better seismic effect and poor thermal insulation effect, the above two measures need to be used together to make the lining having both remarkable thermal insulation effect and seismic effect.

## Conclusions and outlooks

In this paper, with highly damped composite structure combining with lining structure, we proposed a kind of seismic measure for fault-crossing tunnel located in high geothermal area with strong earthquake. We simulated the temperature field and dynamic waves in the finite difference software Flac^3d^, and then we analyzed the thermal insulation and seismic effect from PC to SFRC highly damped composite structure according to the results of temperature, stress and safety factor. Regarding the method and the results, the conclusion are as follows.

1. The study used finite difference method to obtain the required data by the commonly used software in tunnel engineering. The calculation methods are well developed and hold good numerical stability.
2. The incorporation of steel fiber leads to larger factor of thermal conductivity, resulting in higher temperature of SFRC lining, the highly damped composite structure could reduce its influence and lower the temperature of lining.
3. Although SFRC lining have larger stiffness and is better for tunnel supporting, the problem of high stress on lining still needs to be solved. The highly damped composite structure could significantly share and reduce the stress of SFRC lining, also for PC lining.
4. According to the safety factors, the SFRC highly damped composite structure have the best seismic performance while its lining temperature is basically the same with PC lining. The damped structure could enhance the seismic effect when keeping the temperature from rising.

Meanwhile, still some work needs to be done in the future. And we are considering following tests or simulations:

1. More tests for the application of the highly damped composite structure, considering more conditions such as temperature and geologic setting.
2. Further study about the coupling analysis of temperature field and stress field.
3. Further considering the influence of input parameters on uncertain outputs^42,43^, such as the difference between the accuracy of the input parameters and the effect in practical applications.

## Data Availability

The data used to support the findings of this study are included within the article.
